# Two Cases of Giant Uterine Leiomyomas With Myomatous Erythrocytosis Syndrome Treated by Laparoscopic Hysterectomy

**DOI:** 10.7759/cureus.98374

**Published:** 2025-12-03

**Authors:** Noritoshi Aimoto, Takashi Matsumoto, Yonosuke Tsuda, Hosokawa Yumi

**Affiliations:** 1 Gynecology, Osaka Central Hospital, Osaka, JPN

**Keywords:** erythropoietin, erythropoietin producing uterine myoma, myomatous erythrocytosis syndrome, tlh, total laparoscopic hysterectomy

## Abstract

Myomatous erythrocytosis syndrome (MES) is a rare condition characterized by erythrocytosis, the presence of uterine leiomyomas, and the rapid normalization of hematologic parameters following tumor removal. Ectopic erythropoietin (EPO) production by the tumor is considered the main cause.

We encountered two postmenopausal women with giant uterine leiomyomas complicated by MES. Case 1 involved a 21-cm subserosal leiomyoma, and Case 2 involved a 14-cm intramural leiomyoma. We performed total laparoscopic hysterectomy with in-bag morcellation in both cases, enabling safe specimen retrieval. Postoperatively, hemoglobin, hematocrit, and serum EPO levels decreased promptly. Despite the large tumor size, both patients showed only upper-normal serum EPO concentrations, suggesting that tumor weight does not necessarily correlate with EPO secretion.

## Introduction

Uterine fibroids are the most common benign gynecological tumors, occurring in up to 70% of women by age 50 [1]. The primary reasons for treatment intervention are anemia symptoms associated with menorrhagia, compression symptoms caused by fibroids, and dysmenorrhea [2, 3]. However, in rare cases, uterine fibroids may be complicated by polycythemia. Myomatous erythrocytosis syndrome (MES) is a rare tumor-associated condition first reported in 1953. It is defined by three hallmarks: erythrocytosis, histologically confirmed smooth muscle tumors, and rapid normalization of hematologic parameters following tumor resection [4, 5].

We believe the principal mechanism underlying MES is the ectopic production of erythropoietin (EPO) by the leiomyoma tissue itself. Immunohistochemical staining and mRNA analysis in resected tumors support this finding [6, 7]. This unregulated hormone production leads to an overproduction of red blood cells, which significantly increases the risk of thromboembolic events such as stroke and pulmonary embolism. This risk makes diagnosis and treatment crucial even in asymptomatic patients [8].

The standard treatment for MES is surgical excision of the tumor, commonly via hysterectomy. Historically, laparotomy was frequently selected because MES is often associated with giant leiomyomas [5, 7]. However, advances in minimally invasive surgery now allow for laparoscopic management in selected patients, offering reduced morbidity and shorter recovery times [9]. The objective of this report is to present two postmenopausal women with giant leiomyomas complicated by MES, highlight key diagnostic considerations (including preoperative exclusion of leiomyosarcoma in large postmenopausal masses), and demonstrate the safety and feasibility of total laparoscopic hysterectomy with contained morcellation.

## Case presentation

Case 1

A 60-year-old, postmenopausal, nulliparous woman presented with a chief complaint of progressive abdominal distension over several months. Her past medical history was significant for hypertension, which was well-controlled with amlodipine (5 mg/day). On physical examination, we palpated a large, firm, non-tender, and mobile pelvic-abdominal mass, which extended superiorly to the level of the umbilicus.

**Figure 1:**
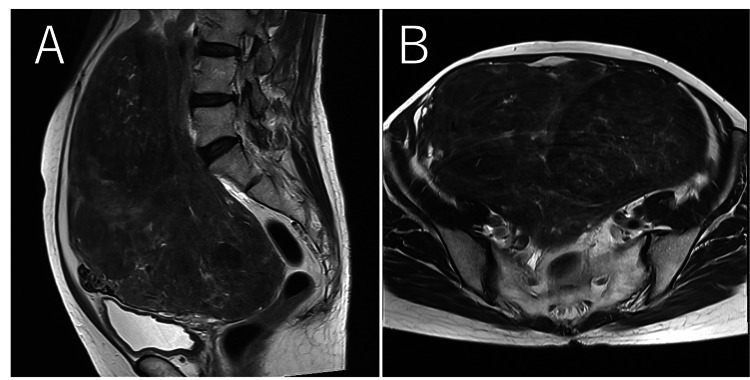
Magnetic resonance imaging (MRI); (A) sagittal T2-weighted image, (B) axial T2-weighted image. A 21-cm mass with low signal intensity was observed, suggestive of uterine leiomyoma. No abnormal signals indicating hemorrhage or necrosis suggestive of sarcoma were detected. No remarkable findings were noted in the adnexal region.

Laboratory investigations revealed marked erythrocytosis, with elevated red blood cell count, hemoglobin, and hematocrit (Table 1). The serum EPO level was within the upper-normal range. To rule out other causes of erythrocytosis, we performed a genetic test for the JAK2 V617F mutation; the test returned negative, effectively excluding polycythemia vera. Abdominal and pelvic imaging, including computed tomography (CT) and magnetic resonance imaging (MRI), showed a 21-cm subserosal leiomyoma without findings suggestive of malignancy, such as heterogeneous necrosis or irregular margins. Given the patient’s postmenopausal status and tumor size, leiomyosarcoma was considered in the differential diagnosis; however, imaging showed no suspicious features (Figure 1).

**Table 1:** Hematologic and erythropoietin parameters in Case 1. The table shows hematologic and endocrine data before and after surgery. Preoperative values were obtained after therapeutic phlebotomy, about one month before surgery. RBC, Hb, and Hct were elevated initially, and all parameters, including EPO, normalized within one month after hysterectomy.

Parameter	At first visit	Preoperative	1 month postoperative	Reference range (unit)
Red Blood Cells (RBC)	612	572	445	370–550 (×10⁴/μl)
Hemoglobin (Hb)	18.5	17.1	13.0	11.5–16.5 (g/dl)
Hematocrit (Hct)	56.3	51.2	41.3	36.0–48.0 (%)
Serum erythropoietin (EPO)	15.3	22.2	9.2	4.2–23.7 (mIU/ml)

The patient initially declined the recommended surgical treatment and opted for therapeutic phlebotomy, undergoing eight weekly sessions of 400 mL blood removal. However, due to the persistence and progression of her abdominal symptoms, she ultimately consented to surgery. We planned a total laparoscopic hysterectomy with bilateral salpingectomy and contained in-bag morcellation for specimen extraction. Although the patient was postmenopausal, she requested ovarian preservation if possible, and we obtained consent for adnexal removal if the surgeon deemed it necessary intraoperatively.

Intraoperatively, we identified a giant leiomyoma arising from the posterior uterine fundus and extending into the right broad ligament. We removed the right adnexa, which adhered to the tumor, while we preserved the left adnexa. We placed the large specimen into a containment bag and morcellated it within the bag before retrieving it vaginally. The total weight of the uterus was 2,470 g. The operative time was 4 hours and 14 minutes, with a blood loss of 25 mL.

**Figure 2:**
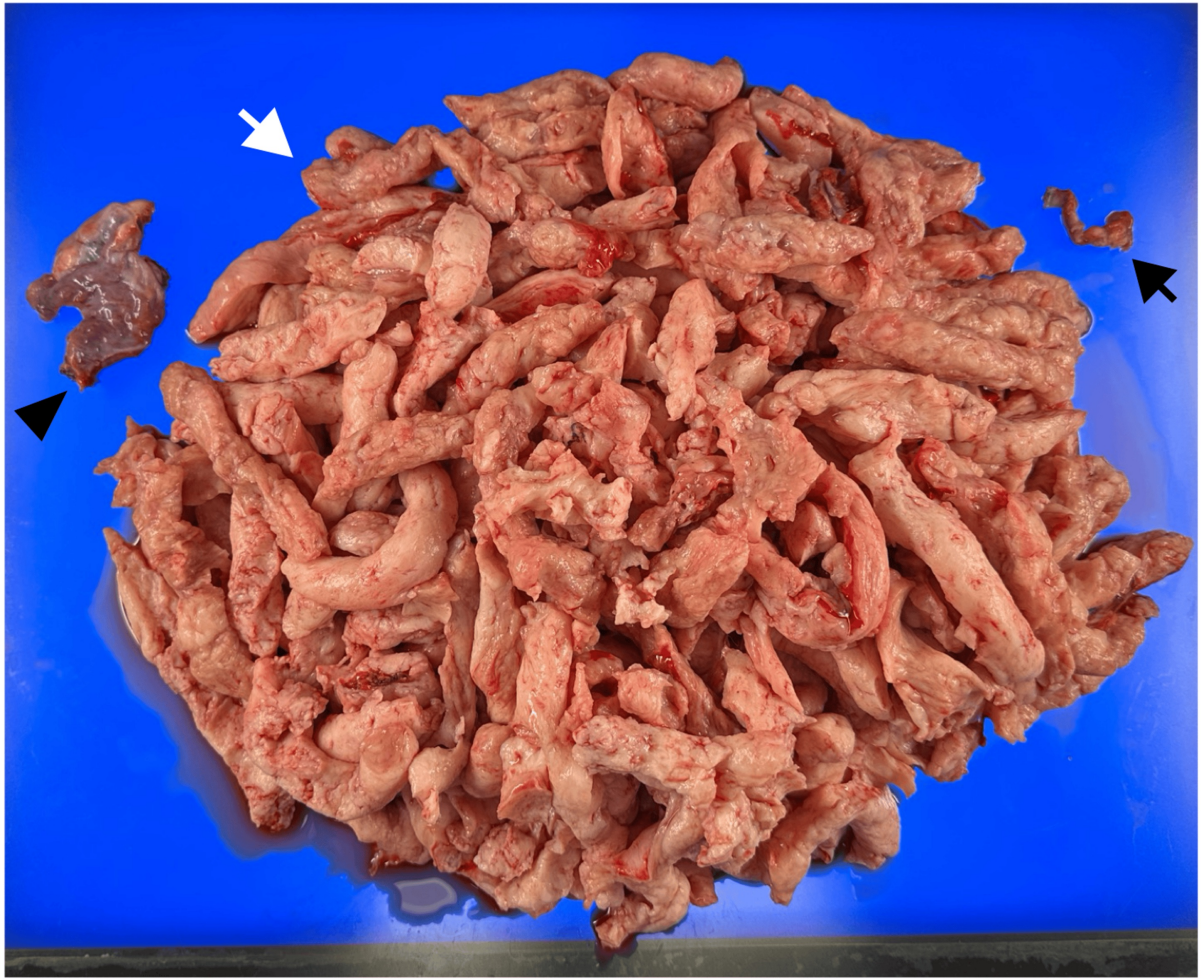
Pathological findings. White arrow: uterus and leiomyoma; black arrow: left fallopian tube; black triangle: right adnexa. No abnormalities were observed in the uterus or adnexa apart from the leiomyoma.

Histopathological examination of the specimen confirmed the diagnosis of a benign leiomyoma, characterized by interlacing fascicles of bland spindle cells (Figure 2). The patient's postoperative recovery was uneventful; she was discharged on the fourth postoperative day in accordance with our institution’s standard postoperative management protocol. At her one-month follow-up appointment, this was confirmed, with her hemoglobin and hematocrit levels returning to normal and her serum EPO level decreasing significantly (Table 1).

Case 2

A 52-year-old, postmenopausal, nulliparous woman presented with worsening abdominal distension. Physical examination revealed a palpable, firm mass in the lower abdomen, consistent with an enlarged uterus.

**Figure 3:**
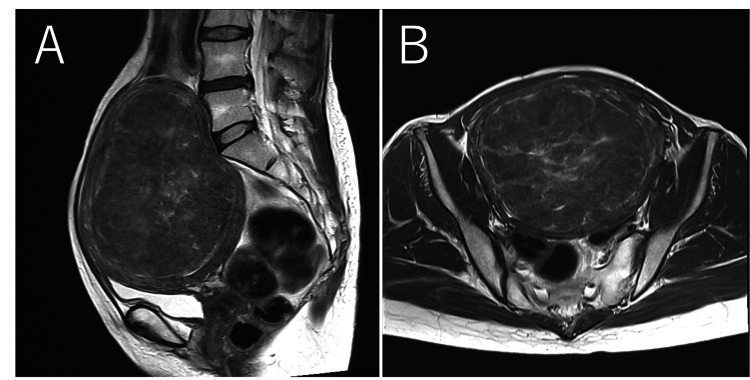
Magnetic resonance imaging (MRI); (A) sagittal T2-weighted image, (B) axial T2-weighted image. No abnormalities were observed in the uterus or adnexa apart from the leiomyoma.

Her initial laboratory results showed significant erythrocytosis (Table 2), while her serum EPO level was in the upper-normal range. Pelvic MRI demonstrated a 14-cm intramural leiomyoma enlarging the uterus. There were no obvious findings suggestive of uterine sarcoma or other malignancy (Figure 3).

**Table 2:** Hematologic and erythropoietin parameters in Case 2. The table shows hematologic and endocrine data before and after surgery. Preoperative values were measured about one month before surgery. Marked erythrocytosis resolved after hysterectomy, with normalization of Hb, Hct, and EPO levels.

Parameter	Preoperative	1 month postoperative	Reference range (unit)
Red Blood Cells (RBC)	576	451	370–550 (×10⁴/μl)
Hemoglobin (Hb)	18.1	13.2	11.5–16.5 (g/dl)
Hematocrit (Hct)	55.8	39.6	36.0–48.0 (%)
Serum erythropoietin (EPO)	18.8	1.9	4.2–23.7 (mIU/ml)

Based on these findings and the diagnosis of MES, we planned a total laparoscopic hysterectomy with bilateral salpingo-oophorectomy. We performed the procedure successfully, placing the specimen in a containment bag for in-bag morcellation and removal. The resected uterus weighed 1,030 g. The operative time was 3 hours and 16 minutes, and the blood loss was 15 mL.

**Figure 4:**
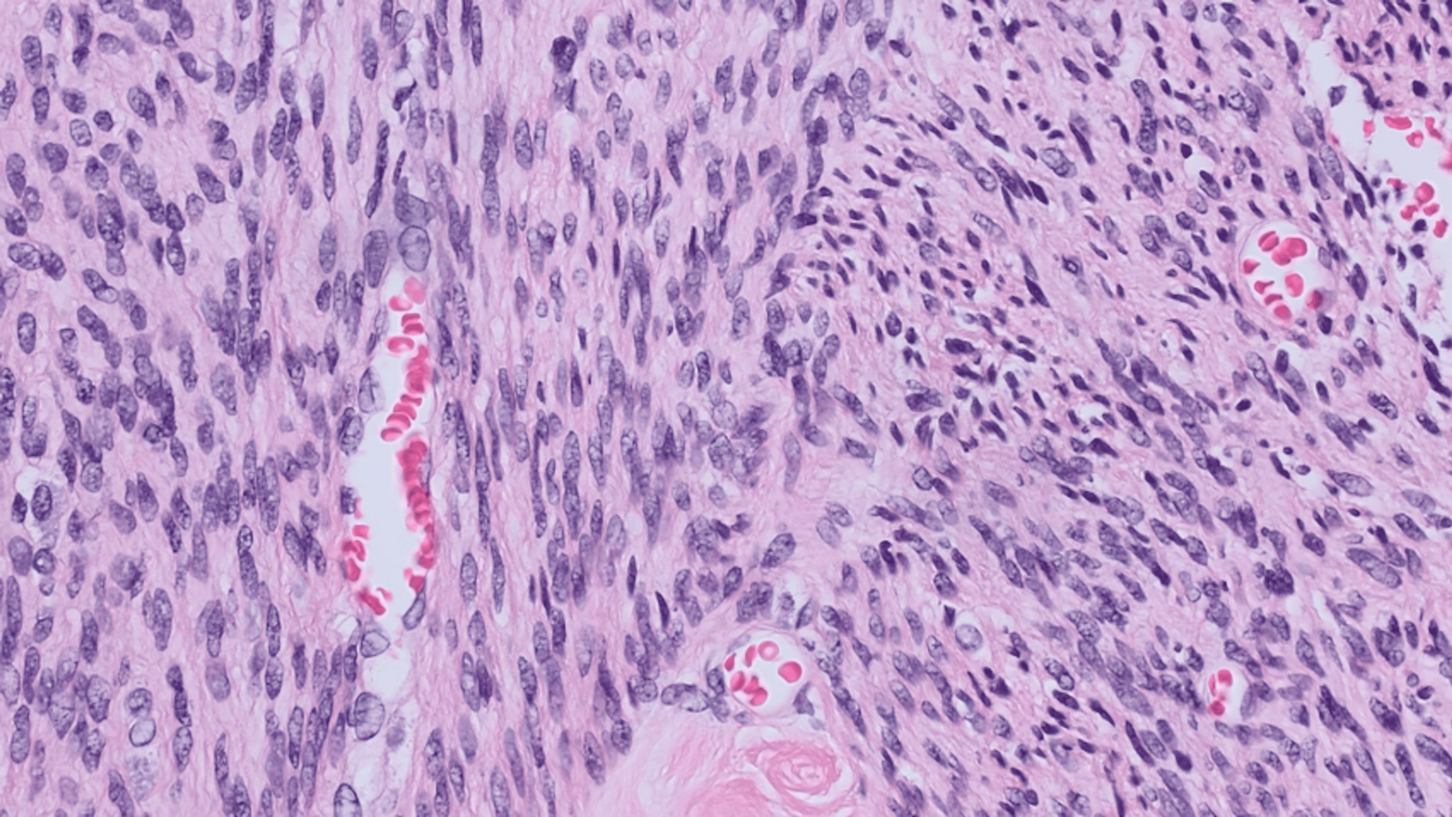
Pathological findings. Fibrosis and degenerative changes were observed, but no tumor necrosis. Histology confirmed leiomyoma.

The final pathology report confirmed a benign uterine leiomyoma (Figure 4). The patient’s postoperative course was smooth and without complications, and she was discharged home on postoperative day 4 in accordance with our institution’s standard postoperative care protocol. As expected, her hematologic abnormalities resolved quickly. At her one-month follow-up visit, her blood counts had completely normalized, and her serum EPO level had decreased to the lower end of the normal range (Table 1).

## Discussion

We diagnosed both patients with myomatous erythrocytosis syndrome (MES) based on the classic triad of a giant uterine leiomyoma, erythrocytosis, and rapid normalization of hematologic parameters following hysterectomy. Although fewer than 70 cases have been documented since 1953, we consider the condition likely underdiagnosed [10].

We consider the ectopic production of EPO by the leiomyoma the leading hypothesis for the pathophysiology of MES [4, 7]. Studies have confirmed EPO mRNA and protein in tumor tissue; expression levels correlate with tumor diameter and vascularity [6]. The prompt postoperative decrease in serum EPO and hemoglobin, as we observed in our cases and others, strongly supports this theory of functional hormone secretion by the tumor [5, 11].

In our cases, despite giant tumors, serum EPO levels remained within the upper normal range. This discrepancy suggests that tumor weight does not directly predict serum EPO levels. Similar inconsistencies have been described: cases with only mildly elevated serum EPO despite positive tumor staining [7, 12], and even a case of a 5,400 g uterus with high serum EPO but low intratumoral EPO [13]. Several explanations exist for this discrepancy. First, a local autocrine/paracrine effect may be at play, as leiomyomas also express EPO receptors; locally produced EPO could be utilized within the tumor for growth and angiogenesis without being released into systemic circulation [12]. Second, a mechanism of secondary renal stimulation could be involved. Tumor compression and congestion may cause local hypoxia, triggering renal EPO production via HIF-mediated pathways. Hypoxic exposure in healthy individuals raises serum EPO within hours, peaking in 1-2 days before normalizing due to feedback [14, 15]. Thus, MES pathophysiology likely involves multifactorial regulation, including local utilization, renal feedback, and systemic dynamics.

Therefore, although elevated serum EPO may support the diagnosis of MES, it does not reliably reflect the severity of erythrocytosis or predict clinical behavior. In our cases, hematologic normalization occurred promptly after tumor removal regardless of preoperative EPO concentrations, indicating that serum EPO has limited clinical utility beyond its diagnostic contribution.

The definitive treatment for MES is surgical removal of the tumor, which typically resolves the erythrocytosis within a week and mitigates the associated high risk of thromboembolism [16]. Historically, laparotomy was the preferred approach for giant leiomyomas. However, ｍore recent evidence demonstrates that total laparoscopic hysterectomy is feasible even for very large uteri, including those exceeding 5,000 g [13]. Moreover, laparoscopic hysterectomy offers shorter hospitalization and reduced pain and infection compared with laparotomy [17, 18]. A major challenge with giant postmenopausal tumors is the preoperative exclusion of malignancy (e.g., leiomyosarcoma). Open power morcellation is discouraged due to the risk of disseminating occult malignant tissue. We successfully addressed this by using in-bag morcellation, a contained extraction technique endorsed by the U.S. Food and Drug Administration (FDA) and American College of Obstetricians and Gynecologists (ACOG) that prevents intraperitoneal spillage and allows for safe minimally invasive surgery [19-21]. Furthermore, given the inherent thrombotic risk of MES, we performed preoperative Doppler ultrasound to exclude deep vein thrombosis, an essential precaution [8].

## Conclusions

We report two cases of MES associated with giant uterine leiomyomas that we successfully and safely treated by total laparoscopic hysterectomy with in-bag morcellation. These cases highlight that serum EPO levels do not necessarily correlate with tumor size. Clinicians should consider MES in the differential diagnosis for patients, particularly postmenopausal women, presenting with giant uterine tumors, and they should confirm the presence or absence of erythrocytosis to guide appropriate management and mitigate thromboembolic risks.
